# Occurrence of microplastics in Russell’s snapper (*Lutjanus russellii*) and associated prey species in the Central Gulf of Thailand

**DOI:** 10.1007/s11356-025-36068-1

**Published:** 2025-02-18

**Authors:** Wanlada Klangnurak, Siriluk Prachumwong, María Belén Alfonso, Haruka Nakano, Suchana Chavanich, Voranop Viyakarn, Suppakarn Jandang

**Affiliations:** 1 Department of Animal Production Technology and Fishery, School of Agricultural Technology, King Mongkut’s Institute of Technology Ladkrabang, Bangkok, 10520 Thailand; 2 Research Institute for Applied Mechanics, Kyushu University, Kasuga-Koen, Kasuga, Fukuoka, 816-8580 Japan; 3 Center for Ocean Plastic Studies, Research Institute for Applied Mechanics, Kyushu University, Chulalongkorn University Research Building 14th floor, Pathumwan, Bangkok, 10330 Thailand; 4 Reef Biology Research Group, Department of Marine Science, Faculty of Science, Chulalongkorn University, Klum Watcharobol Building 3rd Floor, Pathumwan, Bangkok, 10330 Thailand; 5 Aquatic Resources Research Institute, Chulalongkorn University, Institute Building III 9th Floor, Pathumwan, Bangkok, 10330 Thailand

**Keywords:** Microplastic, Lutjanus, Food, Transfer, Contamination, Thailand

## Abstract

Microplastic (MP) contamination in fish may occur via their feeding behavior and ingestion of contaminated prey. This study investigated the presence of MPs in the predator *Lutjanus russellii* (Russell’s snapper) and its prey along the Chumphon coast of the Central Gulf of Thailand. Stomach contents of *L*. *russellii* were analyzed to identify its prey species. Prey species were then sampled from the same geographical area as the predator specimens for subsequent MP analysis. The dietary habits of *L*. *russellii* classify it as a generalist carnivore, consuming a diverse range of food items, including zooplankton, crustaceans, and small fish. No significant correlation was observed between MP abundance and the weight or length of the predator fish (general linear model, *p* > 0.05). Black and red fibers were the predominant MP types in both predator and prey, though MP sizes varied among the sampled species. In predator stomachs, the most common polymers were acrylonitrile butadiene styrene (ABS; 26.32%), polyethylene terephthalate (PET; 21.05%), and polyester (PES; 10.53%). Conversely, prey samples were dominated by PES (17.58%), PET (15.38%), and ABS (13.19%). Notably, similarities in MP characteristics (shape, color, average size, and certain polymer types) were observed between *L*. *russellii* and *Portunus* sp. The detection of smaller PET fibers in *L*. *russellii* compared to *Portunus* sp. (Mann–Whitney *U*-test, *p* ≤ 0.05) suggests the transfer of MPs to *L*. *russellii* through the ingestion of hard-shelled crustacean prey. This study underscores the importance of examining predator–prey interactions to better understand MP contamination pathways in marine ecosystems, particularly in regions like the Gulf of Thailand, where plastic pollution is prevalent. Further research is required to assess the long-term ecological implications of MP transfer within marine food chains.

**Supplementary Information:**

The online version contains supplementary material available at 10.1007/s11356-025-36068-1.

## Introduction

Plastic pollution is a serious global environmental issue, largely caused by poor plastic disposal and waste management, particularly in developing countries like Indonesia, the Philippines, and Thailand (Ferronato and Torretta 2019; Jambeck et al. 2015). Land-based activities, including urbanization, agriculture, and illegal dumping, are major sources of plastic debris (Isobe and Iwasaki 2022; Jambeck et al. 2018). Much of the plastic from terrestrial sources enters the ocean via riverine discharge, a key pathway for transporting plastic waste into marine environments (Lebreton et al. 2017; Thushari and Senevirathna 2020). Once large plastic waste is disposed in the environment, it gradually degrades into numerous smaller particles called microplastics (MPs; ≤ 5 mm) through weathering processes, including UV exposure, wind, and wave action (Kalogerakis et al. 2017; Ter Halle et al. 2016; Thompson et al. 2004).

Recently, MPs have received increasing attention from researchers worldwide. Owing to their small size, light weight, and durability, MPs are found in various environmental components, including soil, water, atmosphere, and detritus (Cole et al. 2011; He et al. 2018; Wright et al. 2020). Recently, MPs have even been detected in human blood (Leslie et al. 2022). Because plastics persist in the environment, they may pose a threat to the health of marine organisms (Alfaro-Núñez et al. 2021). MP ingestion is well-documented in a wide range of marine species, including zooplankton, invertebrates, and vertebrates (Alfonso et al. 2023; Cole et al. 2013; Guzzetti et al. 2018). The complexity of food webs may lead to MP accumulation or transfer across trophic levels, primarily through the ingestion of contaminated prey or water (Costa et al. 2020; Nelms et al. 2018). This can result in MP cycling throughout the ecosystem, affecting a wide range of marine species (Zhang et al. 2019). Because of the similar size ranges of MPs and zooplankton, marine organisms can ingest MPs directly and indirectly, depending on their feeding strategies (Botterell et al. 2019; Day et al. 2024; Wootton et al. 2021a). Moreover, MP polymers, owing to their varying density properties, can either float in the ocean or settle in sediments over time. Their hydrophobic surfaces allow them to adsorb persistent organic pollutants, such as polychlorinated biphenyls and polycyclic aromatic hydrocarbons, from their surroundings (Gateuille and Naffrechoux 2022; Gerdes et al. 2019; Tang et al. 2018). MPs can also bind to heavy metals via other mechanisms (Tang et al. 2022). Consequently, MPs can act as vectors transporting these contaminants through the food web, potentially transferring them to higher-trophic-level consumers (Carbery et al. 2018; Rochman et al. 2013; Smith et al. 2018).

Snapper are among the most important carnivorous fish in marine ecosystems and important commercial fish because of their palatability and nutritional value (Anderson 2002). These fish are found in tropical and subtropical coastal waters (Allen 1985; Luckhurst et al. 2000) and inhabit both marine and brackish environments, enabling them to feed on various prey across diverse habitats (Chukwu and Oyanna 2022). In particular, *Lutjanus russellii* (Russell’s snapper) is a predatory fish that feeds on other fish and crustaceans (Laprise and Blaber 1992) in coastal waters worldwide, including Thailand (the Gulf of Thailand and the Andaman Sea) (Klangnurak et al. 2016). Several studies have documented the ingestion of MPs by the genus Lutjanidae species under natural and experimental conditions (e.g., Bour et al. 2020a, b; Hosseinpour et al. 2021; Pegado et al. 2018).

According to previous studies, the occurrence of MPs in some commercial fish species in Southeast Asia is one of the highest recorded worldwide. It has been speculated that this is due to the density of plastics in this region (Babel et al. 2022; Borges-Ramírez et al. 2020; Susanti et al. 2022). When a marine environment is heavily contaminated with MPs, other secondary producers such as small fish, invertebrates, and zooplankton are likely to be affected, potentially transferring MPs to higher trophic-level organisms in the food chain (Alfonso et al. 2023; Carbery et al. 2018). This study investigated the presence and characteristics of MP contamination in the commercial fish *L*. *russellii* and its prey in the coastal region of Pathio, located in Chumphon Province along the Central Gulf of Thailand. Furthermore, we evaluated the potential trophic transfer of MPs from prey to predator by comparing the shape, color, and polymer types of MPs detected in* L*. *russellii* and its prey, with a specific focus on larger particles identified in the prey.

## Materials and methods

### Study area and sample collection

**Fig. 1:**
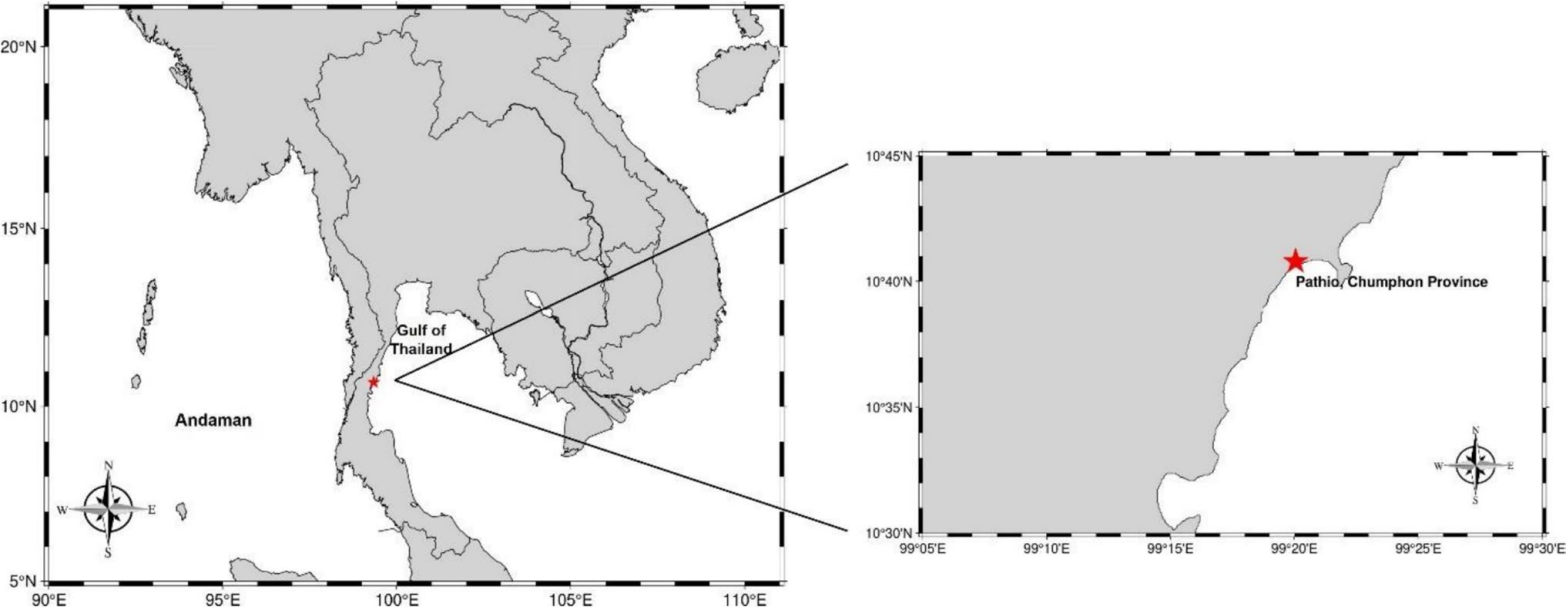
Geographic location of the sampling site on the west coast of the Gulf of Thailand. The map shows a broad view of Thailand (left) and the specific sampling location (right). The red star indicates the sampling point

The Pathio District, located in Chumphon Province along the Central Gulf of Thailand (10.679966°N, 99.334126°E) (Fig. 1), features diverse ecosystems, including estuarine and mangrove habitats. These ecosystems are critical to the livelihoods of the local community, which primarily relies on capture fisheries and coastal aquaculture (Suppanirun 2008). The study site is an important fishing ground for economically significant species, such as pelagic fish (e.g., *Rastrelliger* spp. and *Thunnus* spp.), demersal fish, squid, and crab (Department of Fisheries, Thailand 2018). Commonly employed fishing gear includes gill nets, falling nets, collapsible crab traps, and cuttlefish/squid traps (Petchkamnard et al. 2004; Suppanirun 2008). In addition, the region is interconnected with the Bang Son River, which flows through nearby sub-districts before discharging into the Gulf of Thailand. While this river supports local aquatic ecosystems and livelihoods, it also acts as a potential pathway for litter transport, contributing to pollution in the area. The focal species of this study, Russell’s snapper (*L. russellii*), is a predator fish commonly found in the region. This species occupies various coastal habitats, including mangrove forests, estuaries, and coral reefs, highlighting its ecological importance and prevalence within the study area. To assess the dietary composition of *L*. *russellii*, 121 specimens were captured across two sampling events. The initial sampling, conducted on 6 May 2024, collected 65 specimens, followed by a second sampling on 12 May 2024, which yielded 96 additional specimens. Both sampling events employed hook-and-line capture methods. Notably, only specimens collected during the second sampling were analyzed to assess MP abundance. Based on the dietary content analysis of *L*. *russellii*, all identified prey species were collected from the sampling area using a hand net with a 2-mm mesh size. The prey collection consisted of 175 *Acetes* sp., 65 *Mesopodopsis* sp., 30 *Penaeus* sp., 5 *Harpiosquilla* sp., 23 *Portunus* sp., 30 *Stolephorus* sp., and 20 *Sphyraena* sp. All specimens were stored in a freezer until laboratory analysis.

### Diet analysis

Each fish specimen was measured for fork length (cm) and wet weight (g) (*N* = 161). Each fish was carefully dissected along the mid-ventral line to examine its stomach contents, and the entire digestive system was delicately removed to prevent rupture. The stomach was then opened in a Petri dish to visually assess its content. If no prey items were present, the stomach was recorded as empty. The percentage of empty stomachs was determined by dividing the number of empty stomachs by the total number examined, and multiplying by 100. For stomachs containing prey items, the contents were carefully removed for further analysis. Both the gut and its food contents from each fish were then weighed using an electronic balance. Prey items were observed under a stereomicroscope and classified to the most detailed taxonomic level possible. Food items (*i*) in each dissected specimen were counted to determine the frequency of occurrence (*%F*), numerical percentage (*%N*), and gravimetric percentage (*%W*), according to Hyslop (1980) and Mahesh et al. (2018), as follows:

*Frequency of occurrence (%F)* was interpreted as the presence or absence of each food item across the total number of fish stomachs examined.

$$\%F= \frac{{N}_{i}}{N} \times 100$$

where *Ni* is the number of stomachs containing prey “*i*” and *N* is the total number of stomachs with food.

*Numerical percentage (%N)* refers to the number of occurrences of food item “*i*” in the gut content.

$$\%N= \frac{{N}_{i}}{{N}_{t}} \times 100$$

where *Ni* is the number of food item “*i*” and *Nt* is the total number of food items.

*Gravimetric percentage (%W)* is the weight of each prey item relative to the total weight of gut contents.

$$\%W= \frac{{W}_{i}}{{W}_{t}} \times 100$$

where *Wi* is the weight of food item “*i*” and *Wt* is the total weight of food (gut content).

Additionally, *the index of relative importance* (*IRIi*) (Pinkas et al. 1971) was calculated to determine the importance of each prey item relative to that of others by considering the weight and number of each prey item and the frequency at which each item was found in the stomach.

$${IRI}_{i}=\left(\%N+\%W\right)\times \%F$$

*IRIi* was further expressed as a percentage (Cortés, 1997).

$$\%{IRI}_{i}=\frac{100 \times {IRI}_{i}}{{\sum }_{i=1}^{n}{IRI}_{i}}$$

where *n* is the total number of food items in a taxonomic group.

Finally, to understand the relative level of dietary specialization of the species, *the diet breadth* (*Bi*) was calculated following Novakowski et al. (2008) using Levin’s standardized index formula, which uses the number of occurrences of food items.

$$Bi=\frac{1}{(n-1)}\left[\frac{1}{\left({\sum }_{j}{p}_{ij}^{2}\right)} - 1\right]$$

where *Bi* is Levin’s standardized index for predator *i*, *p*_*ij*_ is the proportion of the diet of predator *i* corresponding to prey *j*, and *n* is the total number of food categories. The level of dietary specialization was classified according to breath niche values into three levels: high (> 0.6), intermediate (0.4–0.6), or low (< 0.4).

### Microplastic extraction

Each fish was measured for wet weight (g) and fork length (cm) (*N* = 96), and then rinsed using Milli-Q water to remove potential MPs attached to the fish surface before dissection. The fish guts were dissected, and food items were removed. The gut of each fish was weighed using an electronic balance. Thereafter, each gut was placed in an individual 100-mL glass jar and preserved by immersion in a 10% neutral buffered formalin solution until MP analysis. Similarly, all prey items were washed three times with Milli-Q water and preserved in 10% neutral buffered formalin.

Sample digestion for MP analysis was performed according to the methods of Klangnurak and Chunniyom (2020) and Cole et al. (2014), with some modifications. Preserved samples were oven-dried at 60 °C for 24 h. The dried samples were then added to a 100-mL glass beaker and immersed in 10 M NaOH at room temperature (28 °C) overnight for digestion, followed by 10 min of ultrasonication at room temperature. Each obtained sample was filtered using polytetrafluoroethylene (PTFE) filter paper (5-µm pore size), employing glass filtration equipment coupled to a vacuum system under a fume hood (Top Lab, Thailand). Subsequently, each filter paper with potential MP particles was placed in a clean glass Petri dish and oven-dried at 60 °C for 4 h to prepare the samples for physical and chemical characterization.

### Physical and chemical characterization of microplastics

All potential MP particles were manually selected using tweezers, placed on a clean Petri dish, and classified under a stereomicroscope (Olympus BX51, Japan). The microscope was equipped with a USB camera (Shodensha DN3V-500, Japan) linked to a computer used to measure and photograph each particle using the Measure Pro light software for digital image processing and size measurement. The number of particles was recorded for each sample in units of particles per individual (particles ind^−1^). All particles were categorized based on their shape, color, and size, following the method established by Free et al. (2014). However, certain particles exhibited unique color combinations, such as red-blue. Therefore, we documented these particles based on their observed characteristics to ensure an accurate representation.

The polymer type of all suspected MPs was identified using a single mercury cadmium telluride detector with the transmission mode of a micro-Fourier transform infrared spectroscopy (µ-FTIR) (Shimadzu AIM-9000 Automated Infrared Microscope, Japan). The wavelength was set between 1300 and 4000 cm^−1^ to avoid wavenumber resolution, and the accumulated scans per sample were set to 4 cm^−1^ and 32 scans. The particle spectra were compared using Shimadzu libraries (standard plastic polymers and thermal- and UV-damaged plastics; see Table S1 for details). Spectra with similarity scores exceeding 70 were considered to be plastic polymer.

### Quality assurance and control

To minimize the risk of contamination during sample collection, transportation, and processing, plastic materials were strictly avoided. The working surfaces, including the working table and fume hood, were cleaned using 70% ethanol. Cotton lab coats and nitrile gloves were worn during the sample preparation and processing. All glassware and tools were washed using tap water and rinsed twice using distilled water before use. Chemical solutions were filtered using a 1.2-μm GF/C filter paper (47 mm) before use. A procedural blank was prepared using identical chemical reagents (NaOH) and underwent the same digestion process as predator (*n* = 3) and prey (*n* = 7) samples. Both the blank and filtered samples on filter papers were placed in 60-mm Petri dishes for visual inspection. No contamination was detected in blanks throughout the experiment.

### Statistical analysis

Results are presented as mean values with standard deviation (SD). A generalized linear model (GLM) was used to evaluate the relationship between the number of ingested MPs and fish weight (g) and length (cm). Given the count nature of the response variable, a Poisson distribution with a log-link function was applied to account for non-negative integer values and potential overdispersion. To test the hypothesis that MP sizes are larger in prey than in predators, we conducted a Mann–Whitney *U* test to compare MP size differences of the same shape and polymer type between predators and prey. However, the color of MPs was not considered in the analysis, as it can be altered during stomach digestion. Statistical significance was determined at *p* ≤ 0.05. Statistical analyses were performed using the Statistical Package for Social Sciences (SPSS) version 28 for Windows and R software (version 3.6.3; R Core Team 2020).

## Results

### Dietary analysis

Of the collected stomachs, 73 (45%) contained food items, while 88 (55%) were empty. Diet analysis (Bi = 0.4) indicated that the predator *L*. *russellii* was a generalist carnivore with moderate dietary specialization. Analysis of the stomach contents revealed eight food items categorized as follows: anchovy (*Stolephorus* spp.), barracuda (*Sphyraena* sp.), shrimp (*Penaeus* sp.), krill (*Mesopodopsis* sp. and *Acetes* sp.), crab (*Portunus* sp.), and mantis shrimp (*Harpiosquilla* sp.). Given that most fish samples were adults, with an average total length of 28.36 ± 4.25 cm, it is unsurprising that their diet primarily consisted of small fish and crustaceans. The main prey items were *Penaeus* sp. and *Stolephorus* spp., which accounted for 47.2% and 36.8% of the *IRIi*, respectively (Table 1). Other food items, except *Harpiosquilla* sp., were compensatory prey items.

**Table 1:** Percentage contribution of different prey items in the overall food components of *L*. *russellii*

Food item	%*N*	%*W*	%*F*	IRI	%IRI
Anchovy: *Stolephorus* spp.	44.7	30.4	29.3	935.9	36.8
Barracuda: *Sphyraena* sp.	22.4	15.4	18.5	307.9	12.1
Shrimp: *Penaeus* sp.	17.1	43.5	27.2	1200.3	47.2
Krill: *Acetes* sp.	5.3	4.5	8.7	44.1	1.7
Krill: *Mesopodopsis* sp.	3.9	2.3	6.5	18.9	0.7
Swimming crab: *Portunus* sp.	4.6	3.5	7.6	31.6	1.2
Mantis shrimp: *Harpiosquilla* sp.	2.0	0.3	2.2	2.7	0.1
Total	**100**	**100**	**100**	**2541**	**100**

*%F* frequency of occurrence, *IRI* index of relative importance, *%N* numerical percentage, *%W* Gravimetric percentage

### Abundance of microplastics in* L. russellii *and prey

This study represents the first assessment of the presence of MPs in *L. russellii* and its associated prey. Microplastics were detected in 14 out of 96 individuals (14.58%) of *L*. *russellii*. The mean MP abundance was 0.20 ± 0.52 particles ind^−1^, with a maximum of 2 particles ind^−1^ recorded. No significant correlation was found between MP abundance and fish weight or length within the sampled population (GLM; *p* > 0.05). The presence of MPs was observed across all examined prey taxa. The highest number of ingested MPs per specimen was recorded in *Harpiosquilla* sp. and *Portunus* sp., with values of 1.2 ± 2.68 particles ind^−1^ and 0.8 ± 1.30 particles ind^−1^, respectively. This was followed by ingestion rates of 0.33 ± 0.66, 0.30 ± 0.57, 0.23 ± 0.63, 0.14 ± 0.38, and 0.09 ± 0.38 particles ind^−1^ in *Penaeus* sp., *Sphyraena* sp., *Stolephorus* spp., *Mesopodopsis* sp., and *Acetes* sp., respectively.

### Microplastic characteristics in* L. russellii *and prey

**Fig. 2:**
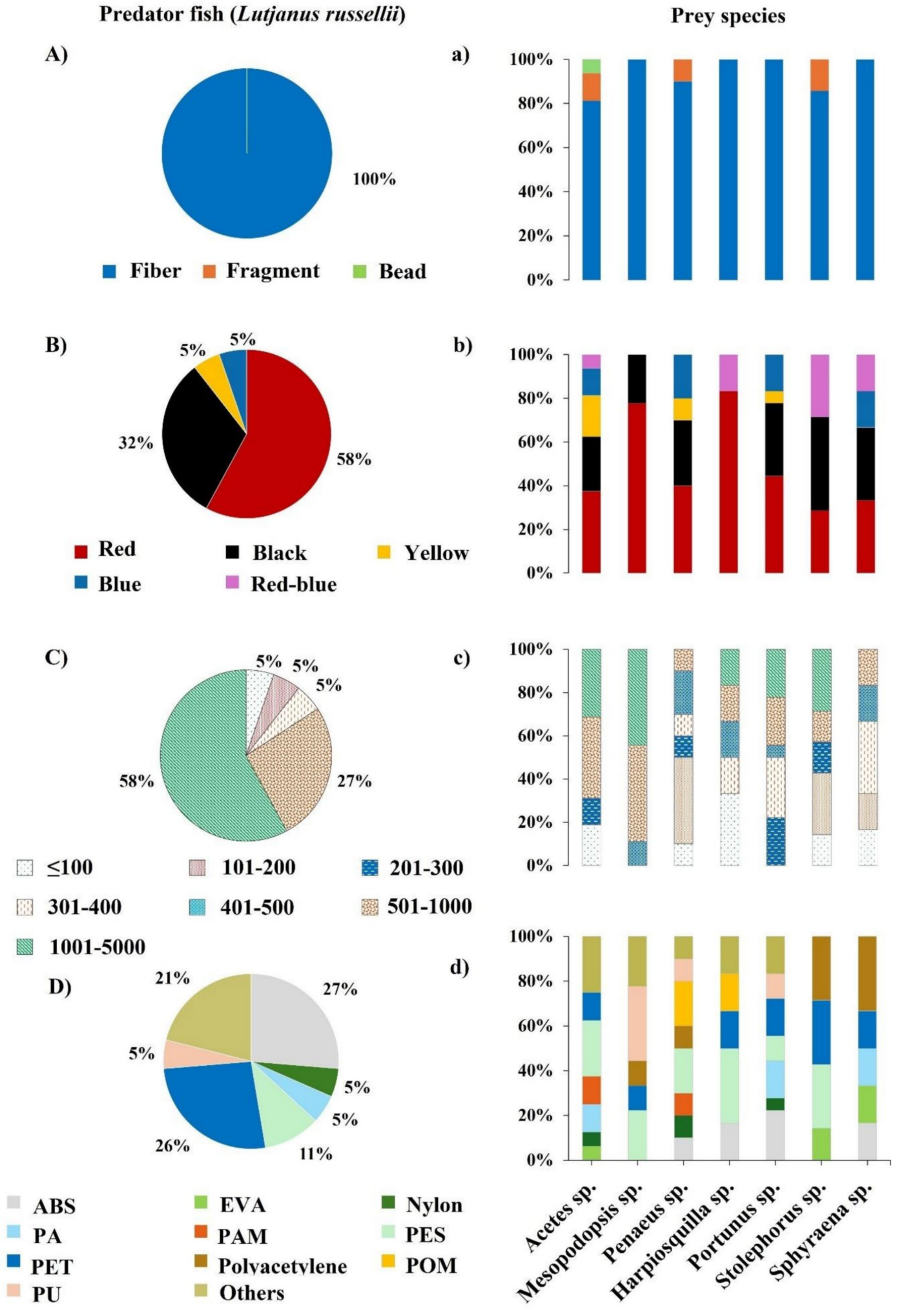
Composition profiles of microplastics (MPs) in *Lutjanus russellii* and its prey species with respect to (A-a) shape, (B-b) color, (C–c) size (μm), and (D-d) polymer type. Uppercase letters correspond to the results, including all species, and lowercase letters to individual species. Abbreviations: ABS (acrylonitrile butadiene styrene), EVA (ethylene–vinyl acetate), PA (polyamide), PAM (polyacrylamide), PES (polyester), PET (polyethylene terephthalate), POM (polyoxymethylene), and PU (polyurethane)

In terms of MP shape categories, fibers were the only shape detected in the gut of *L. russellii* (Fig. 2A). Similarly, prey samples, including *Mesopodopsis* sp., *Sphyraena* sp., *Harpiosquilla* sp., and *Portunus* sp., exclusively exhibited fibers, each with a prevalence of 100%. In contrast, fragments were observed at lower frequencies in some prey species: 12.5% in *Acetes* sp., 10% in *Penaeus* sp., and 14.29% in *Stolephorus* sp. Additionally, beads were detected only in *Acetes* sp., with a low occurrence rate of 6.25% (Fig. 2a). Four primary MP colors were identified in *L. russellii*: red (57.89%), black (31.59%), blue (5.26%), and yellow (5.26%) (Fig. 2B). Prey species demonstrated a similar color distribution, with the addition of a unique red-blue type found in smaller quantities in *Acetes* sp. (6.25%), *Harpiosquilla* sp. (16.67%), *Stolephorus* spp. (28.57%), and *Sphyraena* sp. (16.67%) (Fig. 2b).

The most prevalent MP size ranges detected in *L. russellii* were 1001–5000 µm (57.89%) and 501–1000 µm (26.31%) (Fig. 2C). The size distribution of MPs varied among the prey species. Zooplankton species, such as *Acetes* sp. and *Mesopodopsis* sp., showed similar dominant MP size ranges to *L. russellii*, specifically in the 501–1000 and 1001–5000 µm ranges. In contrast, *Penaeus* sp. showed a 40% prevalence of MPs in the 101–200 µm range, while *Harpiosquilla* sp. displayed a dominant presence of MPs ≤ 100 µm (33.33%). *Stolephorus* spp. had a higher proportion of MPs in the 101–200 µm (28.57%) and 1001–5000 µm size ranges. *Sphyraena* sp. and *Portunus* sp. exhibited a prevalence of MPs within the 301–400 µm range, at 33.33% and 27.78%, respectively (Fig. 2c).

**Fig. 3:**
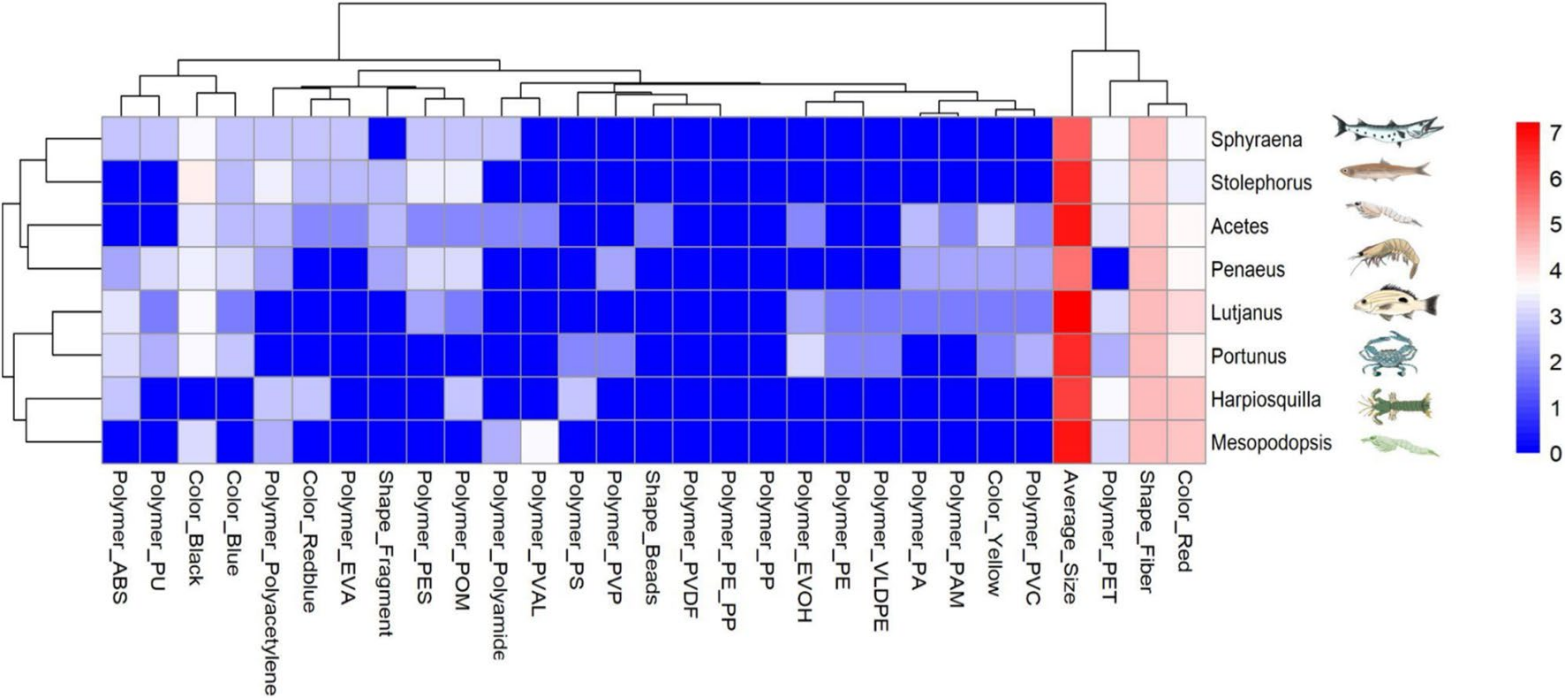
Heatmap illustrating the distribution of microplastic (MP) characteristics across different organisms. The *x*-axis represents various MP properties, including polymer types, colors, shapes, and average sizes, while the y-axis lists the sampled predator fish *Lutjanus* and associated prey species (*Sphyraena* sp., *Stolephorus* spp., *Acetes* sp., *Penaeus* sp., *Portunus* sp., *Harpiosquilla* sp., and *Mesopodopsis* sp.). The color intensity reflects the abundance or frequency of each MP characteristic, with red indicating higher values and blue indicating lower values. The hierarchical clustering on both axes highlights patterns of similarity among organisms and MP characteristics. Abbreviations: ABS (acrylonitrile butadiene styrene), PU (polyurethane), EVA (ethylene–vinyl acetate), PES (polyester), POM (polyoxymethylene), PVAL (polyvinyl alcohol), PS (polystyrene), PVP (polyvinylpyrrolidone), PVDF (polyvinylidene fluoride), PE-PP (PE (polyethylene-polypropylene), PP (polypropylene), EVOH (ethylene vinyl alcohol), PE (polyethylene), VLDPE (very low-density polyethylene), PA (polyamide), PAM (polyacrylamide), PVC (polyvinyl chloride), and PET (polyethylene terephthalate)

According to µ-FTIR analysis, three primary polymer types were identified in the gut of *L. russellii*: acrylonitrile butadiene styrene (ABS) (26.31%), polyethylene terephthalate (PET) (26.31%), and polyester (PES) (10.53%). Additional plastic polymers constituted the remaining 36.84%, including nylon, polyamide (PA), polyoxymethylene (PMP), polyurethane (PU), polyvinylidene fluoride (PVDF), polyvinylpyrrolidone (PVP), and very low-density polyethylene (VLDPE) (Fig. 2D). Among all prey species, 17 polymer types were detected, with five major types dominating: PES (17.58%), PET (16.48%), ABS (13.19%), PA (7.69%), and PU (7.69%), while other polymers accounted for 30.77% of the findings. These additional polymers include ethylene vinyl acetate (EVA), ethylene vinyl alcohol (EVOH), nylon, polyacrylamide (PAM), polyacetylene, polyethylene-polypropylene (PE–PP), PMP, polyoxymethylene (POM), polypropylene (PP), polystyrene (PS), polyvinyl alcohol (PVAL), and polyvinyl chloride (PVC). The predominant plastic polymer in each prey species was identified as follows: PES in *Acetes* sp. (25%); PU in *Mesopodopsis* sp. (33%); PES and POM in *Penaeus* sp. (20% each); PES in *Harpiosquilla* sp. (33.33%); ABS in *Portunus* sp. (22.22%); PES, PET, and polyacetylene in *Stolephorus* spp. (28.57% each), and polyacetylene in *Sphyraena* sp. (33.33%) (Fig. 2d). Overall, similarities in MP characteristics, including shape, color, average size, and certain polymer types, were observed between *L*. *russellii* and *Portunus* sp. (Fig. 3). A clear predominance of high-density polymers (low-density MPs: < 1 g cm⁻^3^, high-density MPs: > 1.12 g cm⁻^3^) was observed in *L. russellii* (63.16%) and in most prey species, including *Acetes* sp. (87.50%), *Mesopodopsis* sp. (88.89%), *Penaeus* sp. (90%), *Harpiosquilla* sp. (100%), and *Portunus* sp. (88.89%). In contrast, a more balanced proportion of low- and high-density polymers were detected in *Stolephorus* sp. and *Sphyraena* sp. (Fig. S1). In addition to non-plastic polymers, natural microfibers ranging in size from 103 to 3512 µm were detected in all samples. The predator, *L. russellii*, contained an average of 0.46 particles ind^−1^. Among the prey species, *Sphyraena* sp., *Portunus* sp., and *Stolephorus* spp. exhibited higher MP counts, with averages of 0.85, 0.83, and 0.43 particles ind^−1^, respectively (Fig. S2).

### Potential transfer of MPs in the food web of* L. russellii*

Among the prey species, only *Portunus* sp*.* displayed a statistically significant difference in the size of larger MPs when compared with predator fish *L. russellii* (Mann–Whitney *U* test, *p* ≤ 0.05, Table S2). No significant differences were observed between the predator and other prey species, except for a few polymers represented by a single particle, which was insufficient for statistical comparison. Detailed analysis of MPs by size, shape, and polymer type indicated the presence of PET fibers in both *Portunus* sp. and *L. russellii*, suggesting the potential transfer of PET fibers within the studied food web (Fig. S3).

## Discussion

### Dietary analysis

Empty stomachs are frequently observed in captured carnivorous fish, especially in field studies (Vries et al. 2020). This occurrence can be attributed to various factors, including species-specific feeding habits, metabolic rates, environmental conditions, and the type of fishing gear used during sampling (Vinson and Angradi 2011). Additionally, fish may regurgitate or expel stomach contents in response to stress during or after capture, a behavior that reduces the contents in their stomachs and may compromise their health and fitness (Zhao et al. 2020). Within the family Lutjanidae, adult fish are often generalist feeders (Anderson 2002; Nanami and Shimose 2013), adapting their diet based on their life stage and habitat. For example, *L. russellii* exhibits distinct dietary shifts throughout its development (Anderson 2002; Iqbal et al. 2023; Madduppa et al. 2013; Nanami and Shimose 2013). Juvenile *L. russellii* primarily consume zooplankton (Chuaykaur et al. 2020), whereas subadults residing in seagrass habitats feed mainly on shrimp (Nuraini et al. 2007). As *L*. *russellii* matures into adulthood, its diet expands to include a wider range of prey, such as smaller fish and invertebrates, including crabs and shrimp (Iqbal et al. 2023; Nanami and Shimose 2013). The dietary patterns observed in the adult fish examined in the present study were consistent with previous observations, further supporting the established knowledge of the species’ feeding habits (Anderson 2002; Nanami and Shimose 2013).

### Abundance of microplastics in* L. russellii *and prey

Plastic pollution is well-documented across marine ecosystems in Thailand, with varying prevalence across locations and ecosystem types (Marks et al. 2020; Prarat and Hongsawat 2022). While high levels of MP accumulation are commonly observed in many coastal areas, Chinfak et al. (2024) reported that the abundance of MPs in Chumphon is two to three times lower than in the coastal area near the Chao Phraya River, Thailand’s primary river system. This difference may account for the lower average MP concentration of 0.2 particles ind^−1^ observed in our study. Although this value aligns with findings for Lutjanidae species along Thailand’s eastern coast (Phaksopa et al. 2021), it is significantly lower—ranging from 2.5 to 75 times—than concentrations reported for Lutjanidae in other regions (Table 2) (Borges-Ramírez et al. 2020; Hamdhani et al. 2024). In addition, stomach fullness is correlated with MP abundance, with fuller stomachs often showing higher MP counts than empty stomachs (Bråte et al. 2016; Liboiron et al. 2016). Conversely, we observed a negative correlation between stomach fullness and MP abundance, likely due to the variability in MP ingestion, retention, and excretion (Vries et al. 2020; Wieczorek et al. 2018). Fish are also known to regurgitate gut contents, including MPs, in response to capture-induced stress (Zhao et al. 2020), lowering MP counts upon examination.

**Table 2:** Abundance of microplastics (in descending order) observed in Lutjanidae in the present study and worldwide

Scientific name	Common name	Location	Weight (g)	MP particles/ind	Target organ	MP shape	MP color	MP size (µm)	MP polymer type	Reference
*Lutjanus madras*	Indian snapper	Rayong, Thailand	47.60 ± 10.50	0.20 ± 0.45	Gut and Gill	Fiber*	Black, Red*	Avg 1770*	PET	Phaksopa et al. (2021)
*Lutjanus russellii*	Russell’s snapper	Chumphon, Thailand	272.18 ± 20.79	0.2 ± 0.5	Gut	Fiber	Red, Black	1001–5000	ABS, PET	This study
*Lutjanus fulviflamma*	Dory snapper	Gulf of Mannar, India	67.70 ± 30.77	0.5 ± 0.12	Gut	Fiber	Blue, White	500–1000	PE, PP	Keerthika et al. (2023)
*Lutjanus* sp.	Snapper	Batangas, Philippines	N/A	0.71	Gut	Filament	N/A	N/A	N/A	Espiritu et al. (2019)
*Lutjanus synagris*	Lane snapper	Isla Grande, Colombia	181.7 ± 45.3	0.9 ± 0.9	Gut	Fragment	Black*	< 5000*	PES*	Jimenez-Cárdenas et al. (2022)
*Lutjanus analis*	Mutton snapper	Brazil’s North Coast, Brazil	N/A	1	Intestine	Pellet*	Clear*	Avg 1820*	PA*	Pegado et al. (2018)
*Lutjanus synagris*	Lane snapper	Brazil’s North Coast, Brazil	N/A	1	Intestine	Pellet*	Clear*	Avg 1820*	PA*	Pegado et al. (2018)
*Lutjanus griseus*	Grey snapper	Campeche Bay, Mexico	428 ± 92	1.25	Gut	Fragment, Fiber*	Yellow	1000–2000*	PTFE, PES*	Borges-Ramírez et al. (2020)
*Lutjanus russellii*	Russell’s snapper	Trat, Thailand	109.66 ± 5.94	1.5	Gut	Fiber	Blue	Avg 1833.33 ± 235.70	PET*	Sutthacheep et al. (2018)
*Lutjanus johnii*	Golden snapper	Pangempang Estuary, Indonesia	160	14 ± 2.0	Gut	Fiber	N/A	N/A	N/A	Hamdhani et al. (2024)
*Lutjanus fulviflamma*	Blackspot snapper	Pangempang Estuary, Indonesia	50	15 ± 2.0	Gut	Fiber	N/A	N/A	N/A	Hamdhani et al. (2024)

^*^Indicates that the study summarizes the overall findings for all fish species examined

*PET* polyethylene terephthalate, *ABS* acrylonitrile butadiene styrene, *PE* polyethylene, *PP* polypropylene, *PES* polyester, *PA* polyamide, *PTFE* polytetrafluoroethylene

MP levels in fish are influenced by multiple factors, including feeding behavior, geographic region, and life stage. For instance, carnivorous fish, such as Lutjanidae, may ingest fewer MPs than omnivorous or planktivorous species because of their selective feeding habits (Zhang et al. 2021). Carnivores tend to target specific prey, which reduces incidental MP ingestion, unlike planktivorous fish that may consume large volumes of particles, thereby increasing their exposure (Aiguo et al. 2022; Hosseinpour et al. 2021; Wootton et al. 2021b). Fish near regions with high plastic accumulation, such as subtropical gyres, exhibit elevated MP levels due to increased environmental concentrations (Boerger et al. 2010; Markic et al. 2018). Body size has also been linked to MP accumulation, with larger fish generally exhibiting higher MP concentrations in many species (Ferreira et al. 2016; Gad and Midway 2022; Pegado et al. 2018). Conversely, we found a negative correlation between body size and MP abundance in *L*. *russellii* (GLM, *p* > 0.05), which is consistent with findings in pelagic and demersal fish from the eastern Gulf of Thailand (Phaksopa et al. 2021) and the western coast of Bangladesh (Matluba et al. 2023). This may reflect species-specific behaviors or environmental factors unique to our study area. Additionally, since our samples were mainly adults, smaller and younger fish may have been more vulnerable to MP pollution, as suggested by Matluba et al. (2023).

Our study detected MPs across all the examined prey taxa, including zooplankton, crustaceans, and fish, revealing pervasive MP contamination in the study area. The highest MP concentrations were observed in benthic crustaceans, which was likely attributable to their sediment-feeding habits. Marine sediments are hypothesized to serve as major reservoirs for MPs and accumulate and retain particles over extended timescales (Mason et al. 2022; Van Cauwenberghe et al. 2015; Zhang 2017). In particular, coastal regions act as sinks for MPs owing to tidal- and wave-driven redistribution, which further entraps MPs within the sediment layers (Woodall et al. 2014; Zhang 2017). MPs from urban runoff, river discharge, and other waste sources settle in these sediments, creating heightened exposure risks for benthic organisms (Peng et al. 2018; Wang et al. 2019). Benthic species, such as *Penaeus* sp., *Harpiosquilla* sp., and *Portunus* sp., which inhabit sediment-rich environments and feed on organic matter within sediments, are especially susceptible to MP ingestion (Williams and Boyko 2016). Crustaceans often indiscriminately consume particles, thereby increasing their MP exposure (Graham and Thompson 2009; Mason et al. 2022; Wang et al. 2019). Consequently, they encounter higher MP concentrations than pelagic species and serve as valuable bioindicators of MP pollution in benthic ecosystems, providing essential insights into sediment contamination levels (Celis-Hernández et al. 2021; Xie et al. 2024).

Zooplankton exhibited the lowest average MP contamination per individual; however, their role as a foundational component of marine food webs raises the ecological concern of low-level contamination. As primary and secondary producers, zooplankton is integral in supporting a wide range of fish and other marine species (Banse 1995; Calbet and Landry 2004). MP contamination in zooplankton poses a risk of MP transfer through the food web, affecting higher trophic levels and potentially leading to bioaccumulation in predator species (Alfonso et al. 2020, 2023; Colen et al. 2020). Moreover, in natural environments, MPs can adhere to or entangle zooplankton appendages, further increasing the likelihood of predator ingestion (Gunaalan et al. 2023).

### Microplastic characteristics in* L. russellii *and prey

The predominance of synthetic fibers as the main shape of MPs in both fish and their prey, as observed in this study, aligns with prior research on marine organisms (e.g., Constant et al. 2022; Piskuła and Astel 2023). This finding likely reflects the high abundance of synthetic fibers in the environment, especially from textile sources (Cesa et al. 2017; Suaria et al. 2016). Given their persistence and frequent interactions with marine species, these fibers are readily ingested (Liu et al. 2022). Prior studies have also noted that MP shape composition can vary with environmental factors, such as tides and currents, with fibers predominating in winter and fragments in the dry season (Li et al. 2021). This variability suggests that seasonal and local dynamics may significantly influence MP exposure patterns.

Red and black fibers were the most common colors among the MPs in *L. russellii* and their prey, consistent with findings from commercial fish in Thailand (Klangnurak and Chunniyom 2020). The high prevalence of red fibers could be attributed to the widespread use of red fishing gear in the study area, whereas black and or other colored fibers may be linked to other synthetic textile sources (Cesa et al. 2017; FAO-ICAC 2013). In contrast, blue fibers are frequently detected in global marine environments, likely originating from gillnets or nylon ropes (Barboza et al. 2023; Wright et al. 2021).

Differences in MP size were also notable between predator and prey species. Predatory fish in this study ingested MPs in the 1001–5000 µm range, while prey species displayed a broader range but contained mostly particles smaller than 1000 µm. This size distribution may reflect species-specific feeding behaviors and the range of particle sizes that each species can ingest (Gouin 2020). The body size of an organism may be related to the size of plastic ingested (Caron et al. 2018; Rummel et al. 2016). Studies have also shown that larger fish such as tuna and mackerel tend to ingest larger plastic particles, possibly because of their gape size and feeding habits (Lopes et al. 2020; Pereira et al. 2020; Yick and Travers 2022). However, the deformability of fibers can facilitate their ingestion, even when they exceed the gapes of the organism (Desforges et al. 2015). Consequently, the relationship between an organism’s size and the prey or MP size it ingests is not strictly linear and varies according to the feeding strategy, dietary preference, and the available MP size range in the environment (Kahane-Rapport et al. 2022; Scharf et al. 2000).

Analysis of polymer types adds another layer to the complexity of MP contamination. ABS, PET, and PES are prevalent in *L. russellii*, and prior research has identified them as high-risk polymers because of their potential toxicity to both marine organisms and humans (Gallo et al. 2018; Yuan et al. 2022). Their presence in prey taxa (e.g., crustaceans, fish, and zooplankton) suggests extensive polymer contamination and the potential for trophic transfer, posing further risks across the food chain (Bajt 2021; Ng and Obbard 2006). As most of the PES and PET in this study were fibers, these particles may have shred from the synthetic textile (e.g., Browne et al. 2011; Cesa et al. 2017; FAO-ICAC 2013). Notably, high-density polymers were more frequently detected in predator fish and benthic prey species than in pelagic prey, which exhibit a more balanced ratio of low- to high-density polymers. This distribution may reflect the feeding habitats of pelagic species, which primarily encounter low-density polymers at the water surface (Lebreton et al. 2018; Nakano et al. 2024), whereas high-density polymers such as PES, PET, and PA are common among benthic organisms (e.g., corals, shrimp, and crabs) owing to their settling patterns (Hossain et al. 2020; Jandang et al. 2024; Zhang et al. 2021).

The presence of non-synthetic fibers, such as natural cellulose fibers, adds a nuanced dimension to the study of MP contamination. While these fibers are often perceived as less harmful than synthetic MPs, their prolonged exposure to marine environments facilitates adsorption–desorption dynamics with toxic substances, including heavy metals (Sharma and Krupadam 2022). Consequently, natural fibers may pose significant ecological risks to marine organisms and ecosystems (Athey et al. 2020; Carbery et al. 2018). This highlights the multifaceted nature of environmental contamination, wherein both synthetic and natural fibers act as vectors for pollutant transmission and contribute to ecological degradation.

### Potential transfer of MPs in the food web of* L. russellii*

This study examined MP transfer from prey to predator fish in a specific foraging area, exploring the complex interactions that may lead to MP accumulation at higher trophic levels. While Justino et al. (2023) suggested that MPs in prey could be transferred to predator fish, our findings revealed wide variability in the physical and chemical characteristics of MPs detected in fish and their prey. Notably, only *Portunus* sp. exhibited a clear potential for the transfer of PET fibers to predator fish, highlighting species-specific factors influencing MP accumulation.

The transfer of MPs from lower- to higher-trophic organisms may be driven by several ecological and physiological factors, including MP concentrations in the environment, feeding behaviors, and digestive processes (Boerger et al. 2010; Nelms et al. 2018; Pan et al. 2019). In this study, prey digestibility emerged as a significant factor. For example, while prey, such as shrimp, fish, and krill, are easily digested and excreted, crabs with hard exoskeletons present more challenges in digestion, leading to the extended retention of MPs. Species-specific digestion rates and gut clearance in organisms may influence opportunities for MP transfer. These crabs are known to retain MPs for prolonged periods (14–21 days, according to Watts et al. 2014), far exceeding the MP retention time of other prey species such as the Japanese anchovy (*Engraulis japonicus*, ~ 20 h) (Ohkubo et al. 2022), the three-spined stickleback (*Gasterosteus aculeatus*, ~ 20–48 h) (Bour et al. 2020a, b), shrimp (*Penaeus* spp. ~ 27–75 h) (Gray and Weinstein 2017), and krill (~ 1 day) (Dawson et al. 2018). This difference in retention time supports the notion that harder-shelled prey may contribute significantly to MP accumulation in predators.

Top predators are particularly vulnerable to MP contamination owing to both direct ingestion and trophic transfer from contaminated prey (Au et al. 2017; Ferreira et al. 2016; Nelms et al. 2018). Fish predators can encounter MPs directly from environmental sources, including water columns and sediments (Lusher et al. 2013; Ory et al. 2018; Roch et al. 2020). Microplastic uptake in fish can occur through both active (e.g., prey ingestion) and passive (e.g., gill filtration) mechanisms (Aiguo et al. 2022; Roch et al. 2020; Ronda et al. 2023). Larger fish, with more developed oral capacities and filtering anatomy, may be especially prone to MP intake through drinking, which further increases their exposure to environmental MPs (Gad and Midway 2022; Lopes et al. 2020; Pereira et al. 2020).

However, this study had some limitations. Although seawater and sediment are potential sources of MP intake by fish and their prey, we did not collect environmental samples, which may limit the generalizability of our findings regarding MP transfer from prey to predators. Additionally, as a predator, *L*. *russellii* collected in this study predominantly ingested small prey. Consequently, the findings may not provide a comprehensive representation of its dietary habits, particularly if larger prey items were also consumed but not detected. Future studies should incorporate a broader range of environmental samples, including seawater and sediment, and consider seasonal sampling to better capture variations in prey availability and MP contamination patterns. This approach would offer a more detailed representation of MP contamination in predatory fish and their associated prey species.

## Conclusion

This study provides the first evidence of MP contamination in predator fish and their associated prey species. The results revealed that the predator *L. russellii*, and all prey species analyzed from Pathio, Chumphon, contained MPs. Notably, the average abundance of MPs in *L. russellii* was lower than that reported globally for Lutjanidae species, despite Thailand being a region with significant plastic pollution. This discrepancy could be explained by factors such as the high proportion of empty stomachs in the sampled fish, differences in fish age, or species-specific sensitivity to MP ingestion. Although variations in MP size were observed among the sampled species, consistent similarities in MP shape, color, and polymer type suggested a highly localized abundance of these MP types in the area. A particularly concerning finding was the potential trophic transfer of PET fibers from *Portunus* sp. to *L. russellii*. Given that PET is classified as a hazardous polymer, this raises significant ecological concerns regarding the risks that MPs in prey species pose to the marine food web. The level of risk, however, may depend on prey type, as digestion and excretion processes vary. For example, hard-shelled crustaceans require longer digestion times, potentially increasing the likelihood of MP accumulation in predators. These findings emphasize the urgent need for targeted mitigation strategies to address MP pollution and call for further research to assess its ecological impact on marine ecosystems.

## Supplementary Information

Below is the link to the electronic supplementary material.

Supplementary file1 (DOCX 512 KB)

## Data Availability

See Supplementary Materials file.
