# Supplementary material for: Occurrence of microplastics in Russell’s snapper (Lutjanus russellii) and associated prey species in the Central Gulf of Thailand
Source: Environ Sci Pollut Res Int. 2025 Feb 18;32(10):5955–70. doi: 10.1007/s11356-025-36068-1 (PMC11913927; doi:10.1007/s11356-025-36068-1)
Supplement: Supplementary file 1 — Supplementary file1 (DOCX 512 KB) [file 11356_2025_36068_MOESM1_ESM.docx]

**Journal: Environmental Science and Pollution Research**

**Occurrence of microplastics in Russell's snapper (*Lutjanus russellii*) and associated prey species in the Central Gulf of Thailand**

Wanlada Klangnurak^1^, Siriluk Prachumwong^1^, María Belén Alfonso^2,3^, Haruka Nakano^2,3^,

Suchana Chavanich^4,5^, Voranop Viyakarn^4,5^ and Suppakarn Jandang^2,3*^

^1^ Department of Animal Production Technology and Fishery, School of Agricultural Technology, King Mongkut's Institute of Technology Ladkrabang, Bangkok 10520, Thailand

^2^ Research Institute for Applied Mechanics, Kyushu University, Kasuga-Koen, Kasuga 816-8580, Japan

^3^ Center for Ocean Plastic Studies, Research Institute for Applied Mechanics, Kyushu University, CU Research Building 14^th^ floor, Bangkok, 10330, Thailand

^4^ Reef Biology Research Group, Department of Marine Science, Faculty of Science, Chulalongkorn University, Pathumwan, Bangkok, 10330, Thailand

^5^ Aquatic Resources Research Institute, Chulalongkorn University, Institute Building No.3, 9^th^ floor, Bangkok, 10330, Thailand

***Corresponding Author:** Suppakarn Jandang

Email address: [suppakarn.j@riam.kyushu-u.ac.jp](mailto:suppakarn.j@riam.kyushu-u.ac.jp)

**Supplementary material**

**Table S1** The library used for Fourier-transform infrared (µFT-IR) analysis in this study

| 1 | Food Additives |
| --- | --- |
| 2 | Inorganic |
| 3 | Organic |
| 4 | Polymer |
| 5 | Trans Food Additives |
| 6 | Trans Inorganic |
| 7 | Trans Organic |
| 8 | Trans Polymer |
| 9 | Thermal damaged |
| 10 | UV damaged |

**
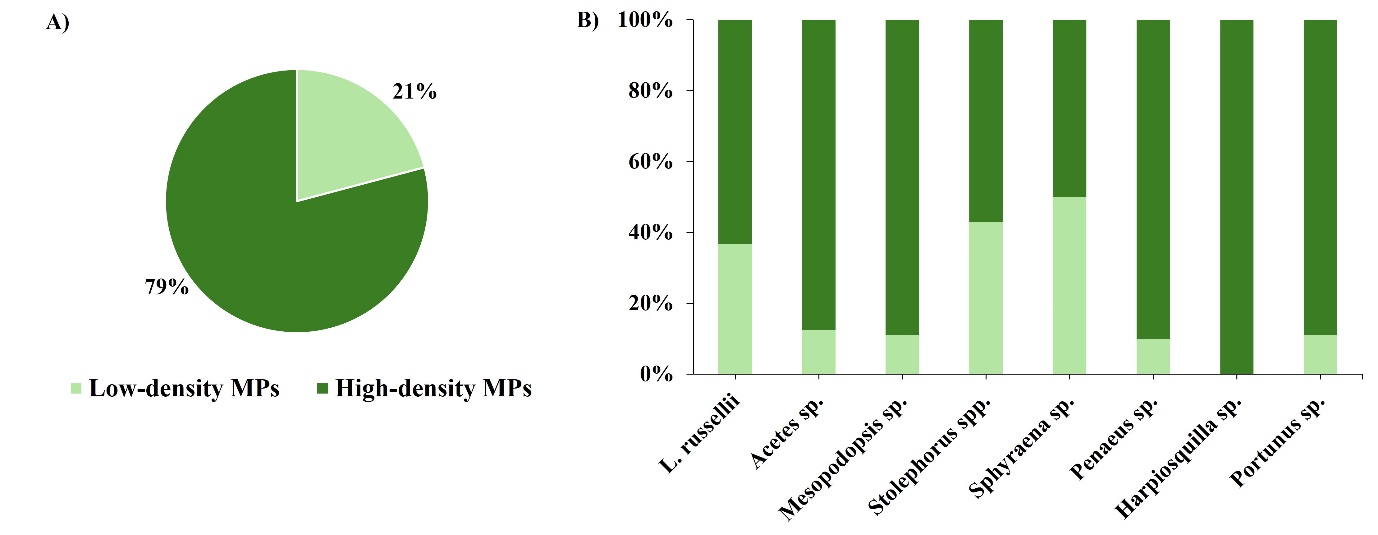
**

**Fig S1** Proportion of low- and high-density microplastics (MPs) with respect to (A) all organism species and (B) individual species


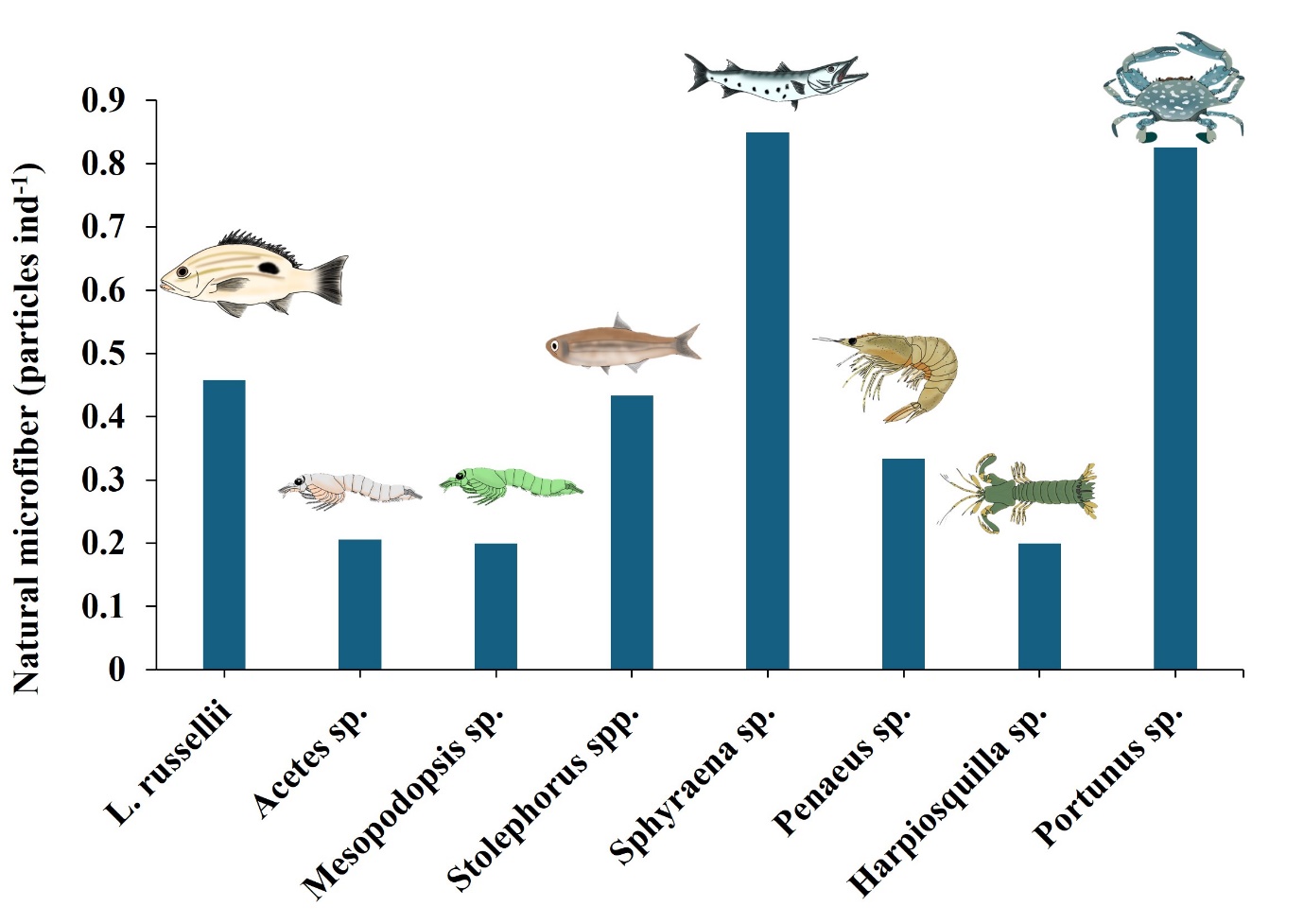


**Fig S2** Mean abundance of natural microfiber per individual in studies of predator fish *Lutjanus russellii* and associated prey

**Table S2** The Mann-Whitney U test was employed to assess potential differences in the presence of microplastics (MPs) between predator (*Lutjanus russellii*) and each prey


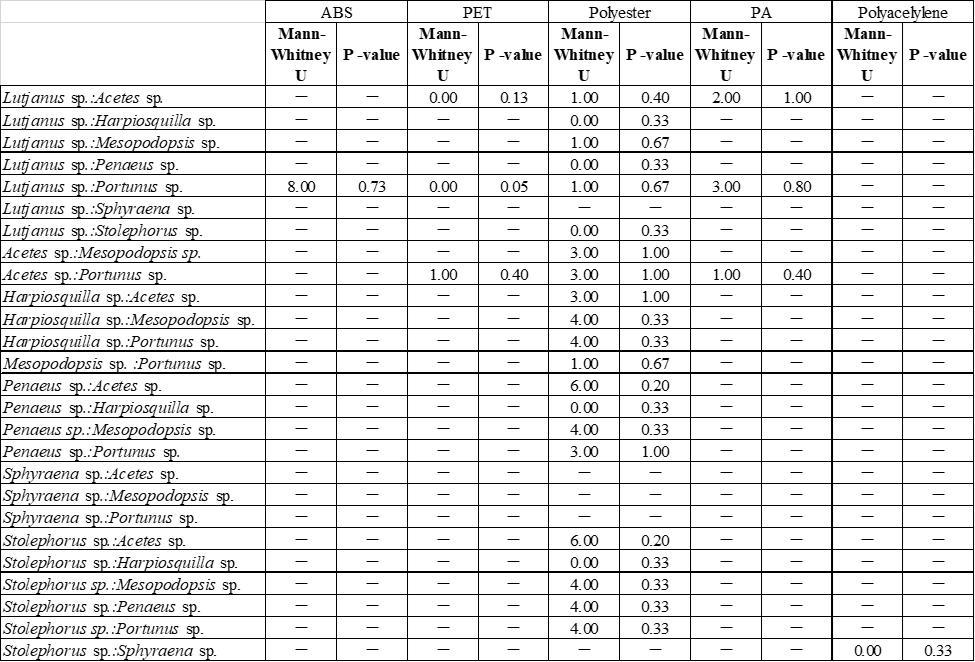


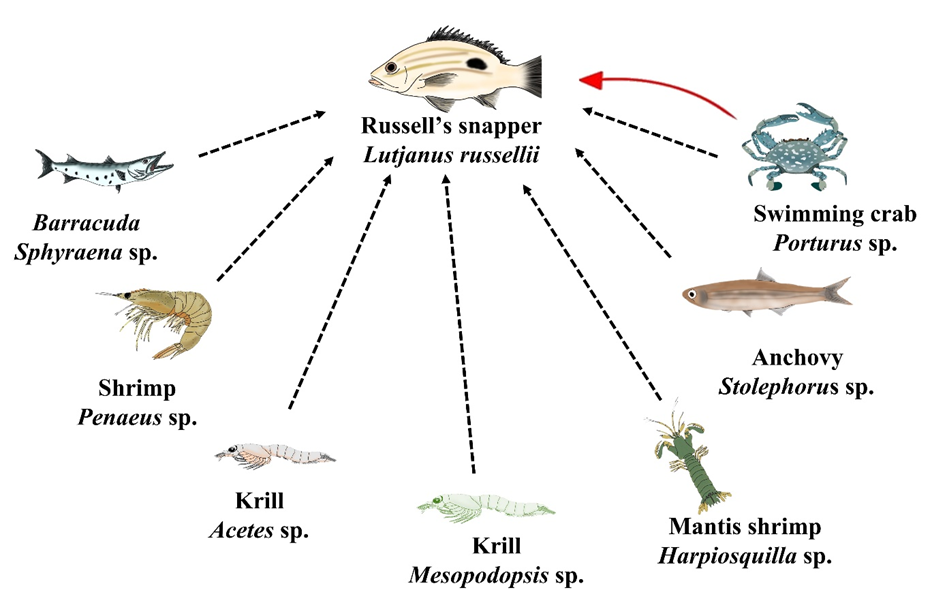


**Fig S3** Diagram illustrating the food web of *Lutjanus russellii* in Pathio, Chumphon, located in the lower Gulf of Thailand. Each organism represents the food consumed by *L*. *russellii*. The red arrow indicates the potential transfer of the Polyethylene Terephthalate (PET) fibers from the crab, *Portunus* sp. to Russell’s snapper *L*. *russellii* (Mann–Whitney U test, *p* ≤ 0.05)
